# Emerging nanomedicines for effective breast cancer immunotherapy

**DOI:** 10.1186/s12951-020-00741-z

**Published:** 2020-12-09

**Authors:** Amirhossein Bahreyni, Yasir Mohamud, Honglin Luo

**Affiliations:** 1 Centre for Heart Lung Innovation, St. Paul’s Hospital, 1081 Burrard St, Vancouver, BC V6Z 1Y6 Canada; 2 Department of Pathology and Laboratory Medicine, University of British Columbia, Vancouver, BC Canada

**Keywords:** Breast cancer, Cancer immunotherapy, Nanoparticle, Tumor microenvironment, Biomaterial, Dendritic cells, Vaccine

## Abstract

Breast cancer continues to be the most frequently diagnosed malignancy among women, putting their life in jeopardy. Cancer immunotherapy is a novel approach with the ability to boost the host immune system to recognize and eradicate cancer cells with high selectivity. As a promising treatment, immunotherapy can not only eliminate the primary tumors, but also be proven to be effective in impeding metastasis and recurrence. However, the clinical application of cancer immunotherapy has faced some limitations including generating weak immune responses due to inadequate delivery of immunostimulants to the immune cells as well as uncontrolled modulation of immune system, which can give rise to autoimmunity and nonspecific inflammation. Growing evidence has suggested that nanotechnology may meet the needs of current cancer immunotherapy. Advanced biomaterials such as nanoparticles afford a unique opportunity to maximize the efficiency of immunotherapy and significantly diminish their toxic side-effects. Here we discuss recent advancements that have been made in nanoparticle-involving breast cancer immunotherapy, varying from direct activation of immune systems through the delivery of tumor antigens and adjuvants to immune cells to altering immunosuppression of tumor environment and combination with other conventional therapies.
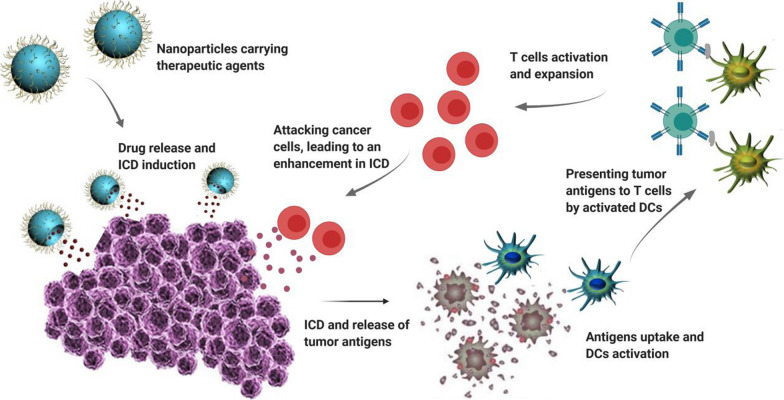

## Background

Breast cancer remains the most frequently diagnosed malignancy and leading cause of cancer-associated deaths among females worldwide [1, 2]. Despite significant declines in mortality, the incidence of breast cancer has risen more than 30% in the last 25 years [3, 4]. Sex, genetic factors, use of hormone therapy, lifestyle and dietary habits, and age are the main risk factors for breast cancer [5].

Breast cancer is a heterogeneous disease, exhibiting various molecular profiles with distinct clinical and biological characteristics [6]. Based on molecular features, breast cancer is classified into 4 common groups: luminal A, luminal B, human epidermal growth factor receptor 2 (HER2)-positive, and basal-like breast cancer [7]. Luminal A and B are hormone (estragon and progesterone) receptor positive, while compared to laminal A, luminal B cancer has higher expression of the cell proliferation marker ki67 and is associated with poor prognosis. Estrogen antagonists like tamoxifen and aromatase inhibitors are the most common drugs used for these two subgroups of breast cancer. For HER2-positive breast cancer, HER2 is overexpressed whereas both estragon and progesterone receptors are negative. Targeting HER2 by specific antibodies such as Trastuzumab is the typical treatment for this type of breast cancer. Basal-like breast cancer is hormone- and HER2-negative (also known as triple-negative breast cancer, TNBC) and considered as the most aggressive breast cancer. Treatment options for TNBC are currently limited. Despite great advancements in therapeutic strategies, ~ 30% of patients with primary breast cancer will ultimately progress to the metastatic stage of this disease [8], and the 5-year survival for metastatic breast cancer is below 30% [9]. Therefore, it is urgent to explore alternative therapeutics to achieve better responses in different breast cancer subtypes.

Immunotherapy has recently changed the concept of cancer treatment [10]. Compared with conventional therapies, immunotherapy has several advantages, including its ability to generate long-lasting memory of antitumor responses [11]. Antigen-presenting cells (APCs) recognize tumor-specific antigens and present MHC-restricted epitopes to T cells to trigger antitumor immune responses [12, 13]. However, tumor cells adapt to escape the immune surveillance [14, 15]. Numerous strategies, including cancer vaccines, immune checkpoint blockades and chimeric antigen receptor T-cell therapies, have been developed to combat against various cancers [16, 17]. However, the efficacy and safety of these approaches remain under debate [18–20].

Biomaterial-based nanoparticles have recently been applied in both pre-clinical and clinical studies to address existing challenges in cancer immunotherapy [21, 22]. Several features of nanoparticles, including great biocompatibility, low side-effect, prolonged half-life, favorable biodistribution, and controlled stimuli‐responsive release of therapeutic agents, make them promising candidates for immunotherapy. Currently, diverse nanomaterials, such as liposomes, microneedle, micelles, dendrimers, protein and polymer-based conjugates and inorganic nanoparticles, are under vast investigations for improving immune responses against tumor [23]. This review aims to summarize and discuss recent findings in nanotechnology-assisted cancer immunotherapy for breast cancer.

## Immune response against cancer

To develop effective cancer immunotherapy, it is vital to understand the interface of the host immune system with tumor cells in the tumor microenvironment (TME). The TME consists of immune and inflammatory cells, blood vessels, neuroendocrine cells, signaling molecules, extracellular matrix, and stromal cells [24, 25]. During each malignancy, alterations in cancer cells mainly due to genetic abnormalities lead to the generation of new antigens. Release of these antigens, as a result of tumor necrosis or apoptosis, triggers activation of APCs. Subsequently, activated dendritic cells (DCs) migrate to the lymph nodes, antigens are presented via MHC I and recognized by helper T cell receptors, leading to stimulation and maturation of B cells and cytotoxic T lymphocytes (CTL). CTLs then move to the TME, followed by destroy of cancer cells and release of additional cancer antigens to boost immune reaction against cancer cells [24, 25]. Interaction between the immune system and cancer cells is divided into three phases, i.e., elimination, equilibrium, and escape [26]. In the elimination phase, cancer cells are recognized and killed by immune system based on the process discussed above. Equilibrium is known as a step that cancer cells have become partially resistant to the immune system. Eventually cancer cells are completely resistant to immune response, causing uncontrolled tumor growth and metastasis in the escape phase.

Multiple mechanisms are involved in tumor cell evasion of the immune system. For example, apoptotic cancer cells release immunosuppressive factors, such as transforming growth factor-β, interleukin-10 (IL-10), and sphingosine-1-phosphate, causing repolarization of M1 to M2 macrophages [27, 28]. Monocyte chemoattractant protein-1 (MCP-1) and bombesin are secreted from dead tumor cells to facilitate monocyte infiltration into the TME [29, 30]. Differentiation of these monocytes into tumor-associated macrophages and infiltration of myeloid-derived suppressor cells (MDSCs) lead to suppression of antitumor immunity [31]. Moreover, tumor cells can evade immune responses by exploiting immune checkpoint pathways, such as the programmed cell death-1 (PD-1) and the CTL-associated protein 4 (CTLA-4) pathways [32, 33]. Overexpression of PD-1 and its ligand (PD‐L1) as well as CTLA‐4 in tumor and/or immune cells prevents T cell activation.

Breast cancer has been considered as a low-immunogenic malignancy, immunotherapy therefore provides a promising treatment option [34–37]. Immune response and distribution of immune cells in breast tumor tissues are quantitatively and qualitatively different based on breast tumor subtypes. The most T cell infiltration and PD-L1 expression can be found in patients with TNBC, while the least infiltration has been reported among patients who are positive for hormone receptors. Infiltration of regulatory T (Treg) cells can be seen in higher grades of malignancy regardless of tumor size and subtype, while elevated expression of CTLA-4 is associated with advanced breast tumor [38]. Currently, immunotherapy has been approved for a subgroup of patients suffering from advanced TNBC; however extensive challenges remain with regard to the safety and efficacy of existing therapeutic strategies.

## Emerging nanoparticle-based systems for cancer immunotherapy

**Fig. 1:**
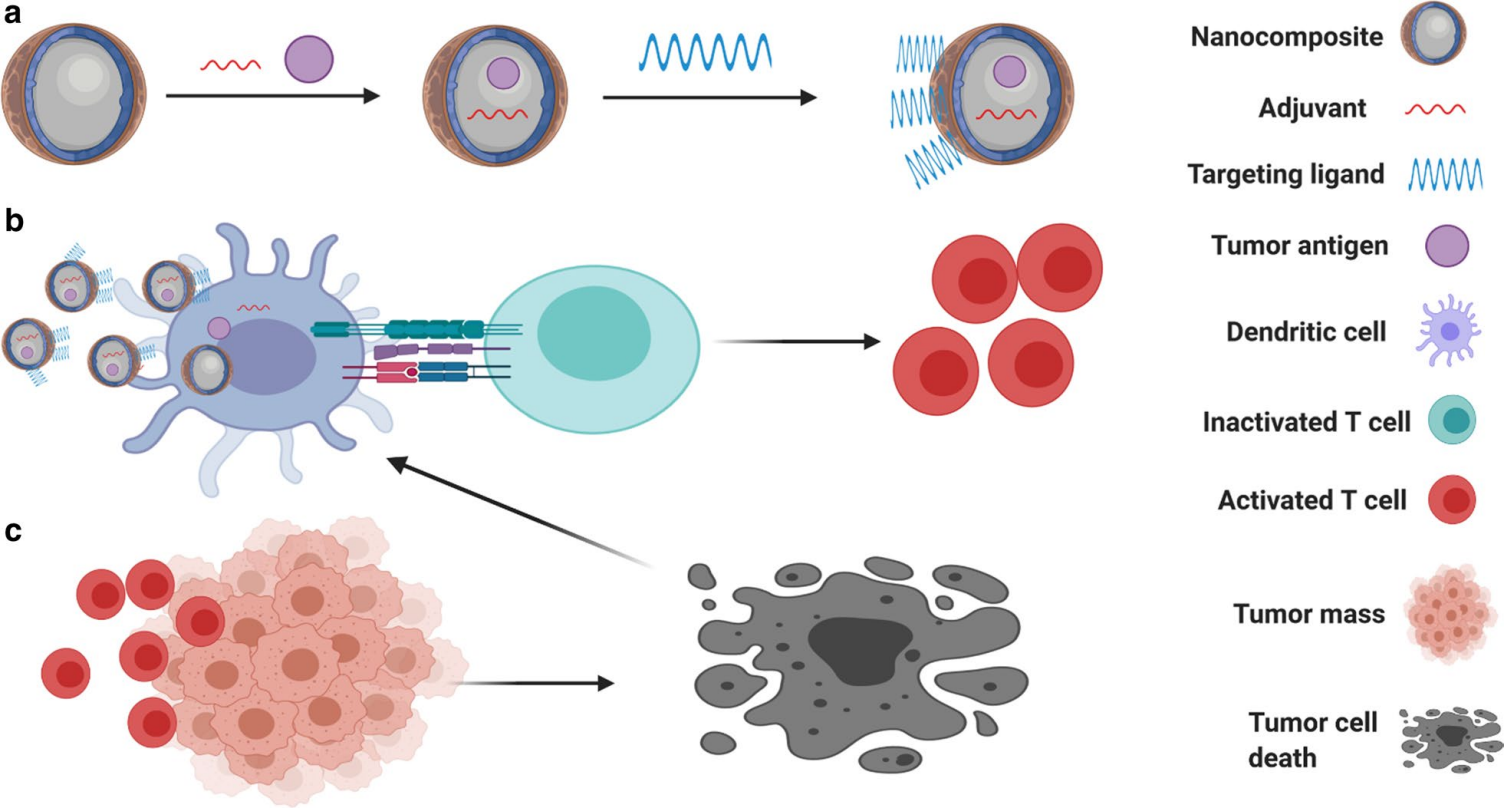
Role of nanoparticles in activation of immune system towards cancer cells. **a** Encapsulation of tumor antigen and adjuvant into nanoparticle, as well as adding targeting ligand on the surface of nanoparticle. **b** Activation of DC through binding of nanoparticles and release of cargo, followed by presenting of tumor antigens to T cells, leading to activation of T cell. **c** T cell response towards cancer cells by releasing Perforin-1, granzyme B and INF-γ, leading to cancer cell death and release of tumor antigens in tumor microenvironment, assisting DC stimulation

Myriad efforts have been made on immune‐modulating system to improve antitumor immunity. Among them, nanomaterials have attracted tremendous attentions due to their ability to overcome limitations in the field of cancer immunotherapy [39]. These nanoparticles are able to convey and release a variety of agents to predetermined areas like APCs with excellent structural stability in serum (Fig. 1). It has been demonstrated that encapsulation of antigens and immunostimulatory molecules into nanoparticles and effective delivery of them into lymph nodes by APCs can significantly promote T- and B-cell responses in comparison with soluble antigens and adjuvants [40]. Moreover, physiochemical characteristics of nanoparticles provide the opportunity of combining different therapies [41–43].

Nanoparticle-based immunotherapy has been investigated in different cancer models. For instance, melittin-lipid nanoparticles have recently been introduced as a novel nanomaterial platform for melanoma immunotherapy [44]. Melittin is the major peptide of European bee venom and has both antitumor and immunoregulatory effects [45]. Compared to free melittin, self-assembled melittin-lipid nanoparticles with no additional tumor antigens or adjuvants are able to facilitate tumor antigen release and initiate enhanced antigen-specific CD8^+^ T cell responses [44]. In vivo study using a melanoma mouse model revealed that melittin-lipid nanoparticles significantly prevent both primary and distant tumor growth, suggesting an excellent candidate to be further developed into a nanovaccine for cancer therapy [44]. IL-12 plays a critical role in T cell activation. A recent study demonstrated that nanoparticles loaded with IL-12 and modified with CD8 and glypican-3 antibodies on the surface are able to interact specifically with CD8^+^ T cells and HepG-2 liver cancer cells, respectively, via the antibody-antigen interactions, leading to the formation of T cell-HepG-2 cell clusters and efficient delivery of IL-12 to CD8^+^ T cells for T cell activation [46]. Cyclic guanosine monophosphate-adenosine monophosphate (cGAMP) is an agonist of stimulator of interferon genes (STING, a pattern recognition receptor) that stimulates innate immunity to promote tumor immunogenicity. A polymerosome with pH-responsive membrane loaded with guanosine monophosphate-adenosine monophosphate (cGAMP) was recently constructed and in vivo delivery of this nanoparticle in mice revealed significant inhibition of melanoma growth, improved tumor response to immune checkpoint inhibitors, and enhanced immunological memory [47]. CpG oligodeoxynucleuotides (ODNs) are synthetic DNA molecules containing CpG motifs that trigger cellular immune responses via toll-like receptor 9 (TLR9) to exert its antitumor action. However, in vivo application of CpG ODNs is limited due to poor stability. Munakata and colleagues [48] formulated a lipid nanoparticle enclosing type-A CpG ODNs and demonstrated that either intratumoral or intravenous administration of this nanoparticle considerably suppresses tumor growth in a mouse model of colon cancer through a CD8^+^ T cell-dependent manner. Furthermore, lipid nanoparticles have been utilized as carriers for delivery of antigen-coding mRNA to DCs. It was reported that vaccination of a lymphoma mouse model with DCs pre-treated with lipid nanoparticles containing an ovalbumin (antigen peptide)-encoding mRNA increases ovalbumin-specific CTL activity and inhibits tumor growth [49].

While most studies have focused on designing nanoparticles to target DCs based on receptor-mediated endocytosis [50–54], macropinocytosis also showed favorable results for nanoparticle internalization. Zhuo et al. [55] reported that surface modification with apolipoprotein E3 significantly enhances the uptake of nanoparticles into DCs mainly via the macropinocytotic pathway and in vivo administration of these modified nanoparticles loaded with imiquimod (an agonist against TLR7) and ovalbumin prevents lung tumor metastasis and has a significant treatment benefit when combined with an anti-PD-1 antibody in B16 melanoma-bearing mice. Efforts have also been made to generate nanoparticles that are able to carry multiple immunostimulatory agents. Nguyen and coworkers [56] fabricated a dual-scale cancer vaccine which is composed of ovalbumin- and CpG ODN-loaded mesoporous silica nanoparticles and mesoporous silica microrods that were incorporated with granulocyte–macrophage colony-stimulating factor (GM-CSF, a DC-recruiting chemokine). It was shown that this hybrid vaccine significantly inhibits melanoma growth and a combination therapy of this vaccine with an immune checkpoint inhibitor produces a synergistic effect against tumor growth [56].

**Fig. 2:**
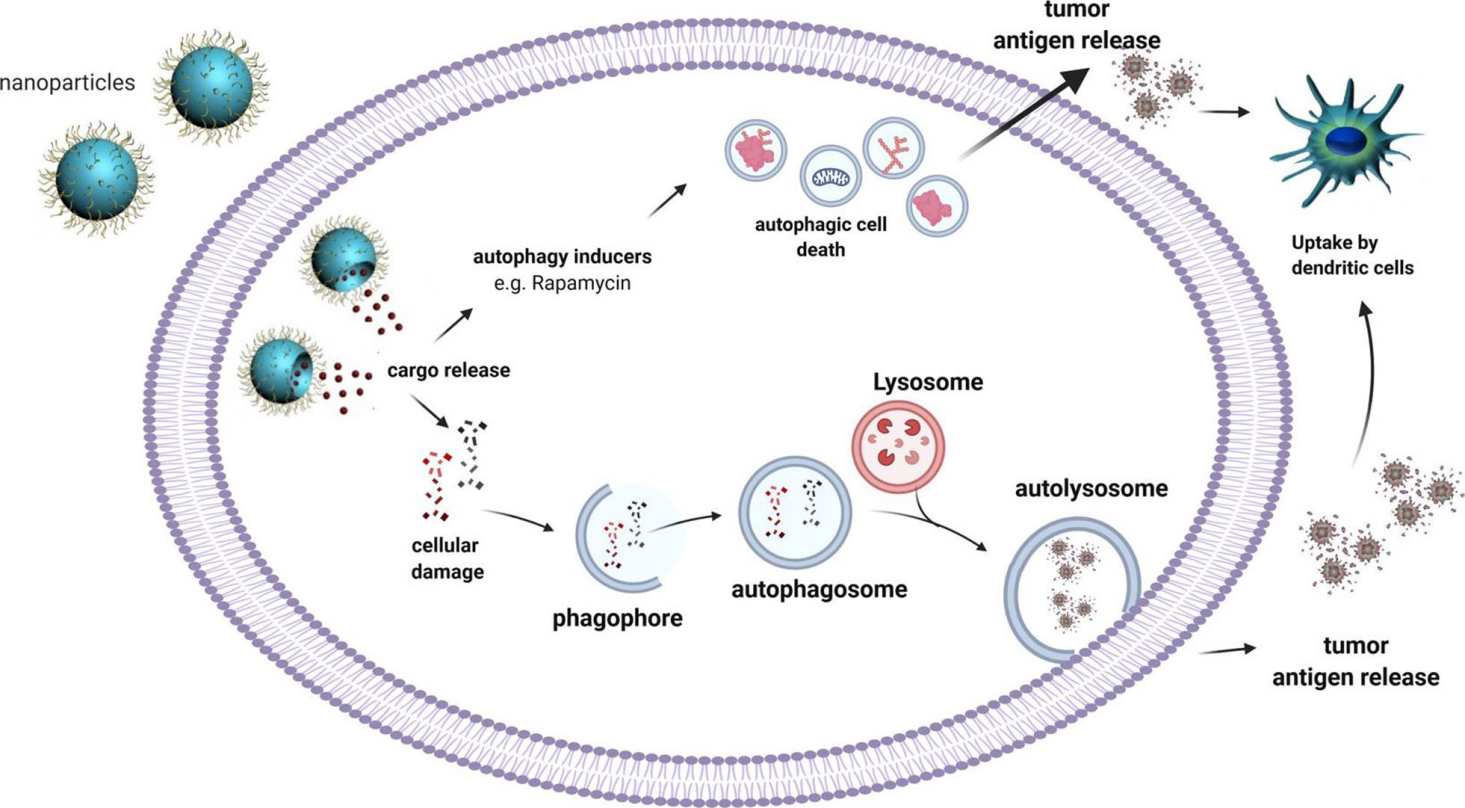
Nanoparticles mediate autophagy-cell death and tumor antigens release into the tumor microenvironment, leading to an enhancement in the DCs recruitment

Autophagy is an evolutionarily conserved self-digestive process and plays a critical role in the maintenance of cellular homeostasis. Emerging evidence also suggests a key role for autophagy in regulating cellular immune responses [57]. Tumor-derived autophagosomes have been applied as a vaccine against cancer cells by enhancing the expression of MHC class I and/or MHC class II on the surface of DCs and B cells [58]. Studies also revealed that nanoparticle-based strategies are effective in autophagy modulation that participates actively in cancer immunotherapy (Fig. 2). For example, induction of autophagy by a doxorubicin-nanoplatform has been shown to promote the activation of DCs through emitting antigens and damage-associated molecular patterns [59]. It was also uncovered that nanoparticle-mediated delivery of oxaliplatin, a chemotherapeutic prodrug and a strong autophagy inducer (STF-62247), triggers robust autophagic death in cancer cells, followed by significant release of tumor antigens in CT26 colon tumor-bearing mouse model [60]. Taken together, these specific examples of novel delivery systems have validated the importance of nanoparticles in achieving effectual cancer immunotherapy.

## Nanoparticles in breast cancer immunotherapy

Both naturally derived and synthetic nanoparticles have been extensively employed in breast cancer treatment due to their excellent compatibility and physiochemical features. To achieve robust immune response against cancer cells, it is vital to deliver effectual amounts of immunostimulatory agents and tumor antigens into immune cells. However, degradation by human enzymes and insufficient transfer of tumor antigens and adjuvants into desired regions like lymph nodes and APCs are the main challenges of typical cancer immunotherapy. Until now, various nanoparticle systems have been developed to overcome these obstacles and to boost immune responses towards breast cancer (Table 1).

**Table 1:** Nanoparticles, assisting immunotherapy in breast cancer

Biomaterial	Cargo	Effects	Refs
Lipid-based			
Liposome	Ursolic acid	Inhibition of STAT5 phosphorylation and IL-10 secretion	[64]
Liposome modified with PEG	Cyclic diguanylate monophosphate and monophosphoryl lipid A	Increased number of APCs and NK cells	[62]
Liposome	cGAMP	Conversion of M2-like phenotype towards M1-like phenotype, enhancement of MHC and costimulatory molecules	[61]
Liposome	Paclitaxel, thioridazine and HY19991	Infiltration of CD4 + and CD8 + T cells into the tumors and consequent attacking of CSCs	[97]
Nanoliposome	Multi-epitope peptides derived from cancer cells	Improved cytotoxic T cell responses and production of IFN-γ	[70]
Lipid calcium phosphate modified with mannose	MUC1 mRNA	Induction of a strong, antigen-specific, in vivo cytotoxic T lymphocyte response against TNBC	[74]
Lipid nanoparticle	Colony-stimulating factor 1 receptor and mitogen-activated protein kinase inhibitors	Increased M1-like phenotype at tumor microenvironment	[69]
Cationic lipid-assisted nanoparticles	Lactate dehydrogenase A-siRNA	Neutralized tumor pH and increased infiltration of CD8 + T and NK cells	[80]
Polymer-based Protein/polysacharide based			
PBAEs	cyclin-dependent kinase 5—CRISPR-Cas9	Downregulation of PD-L1 expression	[76]
PEG-chitosan-lactate	A2 adenosine receptor	Blockage of PKA/CREB signaling pathway, leading to Treg inhibition	[78]
Chitosan-lactate	CD69-specific siRNA	Generation of inflammatory cytokines such as IFN-γ and IL-17	[79]
PLGA coated with human cancer cell membrane fractions	–	Enhanced CD8 + and CD4 + T-lymphocyte populations	[72]
PLGA	CpG coated tumor antigen	Increased expression of CD80/86 and elevated secretion of IL-12	[73]
PLGA-b-PEG modified with triphenyl phosphonium	Zinc phthalocyanine	Release of tumor antigens and thereby activation of DCs, and overexpression of IFN-γ	[96]
Albumin	doxorubicin and T780	Activation of T cell-mediated antitumor immune response and induction of ICD	[89]
Inorganic			
Gold nanoparticle	Ganoderma lucidum polysaccharide	Activation of DCs, enhanced cytokine production and proliferation of CD4 + and CD8 + T cells in splenocytes	[65]
Layered double hydroxide nanoparticles	Indocyanine green, doxorubicin, and CpG	Eradication of primary tumor and prevention of tumor recurrence and metastasis	[87]
Copper sulfide nanoparticles modified with maleimide-PEG	–	Creation of tumor immunogenetic microenvironment, followed by enhancement in the number of tumor-infiltrating CD8 + T cells	[93]
Hybrid nanoparticle			
Fe_3_O_4_ nanoparticles with reduced-graphene oxide (rGO) and PEG	–	Induction of DC activation and ICD in tumor draining lymph nodes	[92]
Albumin coated aluminum hydroxide oxide	Melittin and chlorin e6	Increased generation of reactive oxygen species and consequent ICD	[85]
Naturally derived			
Viral capsid VP2 protein	Multi-neoepitopes including Tmtc2, Gprc5 Qars, and surviving	Enhanced proliferative responses of CD8 + and CD4 + T lymphocytes and generation of granzyme-B in lymphatic nodes local to the tumor	[106]
EVs from NK-92MI cells	IL-15	Increased cytotoxicity against cancer cells	[111]
Lambda phage coat protein gpD	AE37 peptide	Generation of robust immune responses in TUBO model of breast cancer	[108]

### Delivery of immunomodulators using nanoparticles

Since its introduction, liposome, which resembles structure of cell membrane, has been used as a promising nanoparticle. Its ability to conjugate both hydrophilic and lipophilic molecules and its superior structural properties make it an appropriate carrier of numerous agents. Research has revealed that liposome-mediated delivery of cGAMP activates the STING pathway and provokes immune responses towards tumor cells. As a result, tumor growth is suppressed and secondary tumor formation is inhibited in the PD-L1-insensitive mouse models of TNBC [61]. To further boost immune activity in tumor, both STING agonist cyclic diguanylate monophosphate and TLR4 agonist monophosphoryl lipid A were loaded into liposomes, followed by further modification of the liposomes with polyethylene glycol to improve solubility [62]. Systemic application of these developed liposomes increases the number of APCs and natural killer (NK) cells in the blood and tumor, leading to significant inhibition of tumor growth and metastasis in a murine model of metastatic TNBC [62]. Similar to STING, retinoic acid-inducible gene I (RIG-I) is also a pattern recognition receptor, which is activated by viral RNAs. Delivery of a synthetic RIG-I agonist by amphiphilic diblock copolymers has been shown to trigger apoptosis and pyroptosis (an inflammatory form of programmed cell death) and induce upregulation of lymphocyte-recruiting chemokines and type I interferons, followed by prevention of tumor growth and metastasis in mice harboring 4T1 mammary tumors [63].

Ursolic acid (UA) is a natural compound present in many plants. It has antitumor effects through regulating several signaling pathways, including the STAT pathway. However, its application is limited due to poor solubility and stability. Zhang et al. [64] showed that formation of UA crystalline structures inside the liposomes enhances its half-life in circulation and in vivo application of UA-liposomes reduces the populations of MDSCs and Tregs within TME and inhibits tumor growth in the 4T1 breast cancer mouse model. Additionally, incorporation of the immunomodulator polysaccharides isolated from natural herb into nanocomposites was found to activate DCs and enhance cytokine production and proliferation of CD4^+^ and CD8^+^ T cells, and a combination of these nanoparticles with chemotherapeutic doxorubicin significantly inhibits 4T1 tumor growth and lung metastasis without evident toxicity [65].

IL-10 is a key cytokine responsible for tumor immunosuppression in TME. Shen et al. [66] designed a lipid-protamine-DNA nanoparticle loaded with a plasmid that encode an IL-10 trap protein. Application of this nanoparticle enhances infiltration of CTL and inhibits tumor growth in the 4T1 breast cancer mouse model [66]. Granzyme B is a serine protease released by CD8^+^ T cells and NK cells during cellular immune response and an effector enzyme responsible for cytotoxicity [67]. Qian and colleagues [68] generated a nanoparticle-based system for the delivery of granzyme B to tumor tissues to mimics the functionality and outcome of CD8^+^ T and NK cell activation. It was shown that release of granzyme B induces apoptosis and leads to tumor suppression in the MDA-MB-231 breast cancer mouse model [68]. Finally, Ramesh et al. [69] reported that a lipid nanoparticle loaded with inhibitors of colony-stimulating factor 1 receptor and MAPK pathways increases anti-tumorigenic M1-like phenotype at TME and significantly suppresses tumor growth in the highly aggressive 4T1 breast cancer model.

### Delivery of tumor antigens through nanoparticles

With respect to tumor antigen delivery to APCs, it was shown that treatment with tumor cell-derived long, multi-epitope peptides induces more robust immune responses in comparison with short-length peptides. Administration of the nanoliposomes conjugated with a long multi-epitope peptide derived from HER2/neu and a Pan HLA-DR epitope (PADRE) peptide improves CTL responses, and subsequently causing significant inhibition of tumor growth and metastasis in HER2^+^ TUBO mammary tumor-bearing mice [70]. Razazan et al. [71] also reported the development of a liposome vaccine encapsulating Gp2 (a MHC class I peptide derived from HER2/neu) and monophosphoryl lipid A. After vaccination, they observed enhanced antitumor immunity, decreased tumor size, and longer survival time in a breast cancer mouse model overexpressing HER2/neu.

Poly-(D, L-lactide-co-glycolide) (PLGA) is one of the best studied biodegradable polymers. Jin et al. [72] fabricated PLGA-based nanoparticles coated with human cancer cell membrane fractions and reported that these nanoparticles selectively target cancer cells due to the presence of intact membrane-associated proteins including CXCR4 and CD44. Intravenous injection of designed nanoparticles leads to decreased tumor growth and metastasis in breast tumor-bearing mice via disrupting cancer cell-stromal cell interactions and inducing immune responses [72]. A similar study constructed a PLGA-based nanoparticle encapsulating CpG ODN-coated tumor antigen. This nanomedicine significantly enhances both maturation and activation of DCs and inhibits tumor growth and angiogenesis through induction of potent CTL responses in 4T1 breast tumor-bearing mice [73]. Besides tumor peptides, delivery of tumor-associated mRNAs also exhibits a promising enhancement in immune system response. Liu et al. [74] proved that delivery of mRNA encoding tumor antigen MUC1 to DCs by lipid-calcium phosphate nanoparticle initiates a strong, antigen-specific CTL response, resulting in a significant inhibition of 4T1 tumor growth. Modification of developed nanoparticles with mannose, a ligand for mannose receptors that are expressed on DCs, enhances their internalization into DCs, and thereby leading to higher expression of tumor antigen [74].

### Silence or knockout of genes involved in tumor growth by nanoparticles

RNA interference and CRISPR-Cas9 genome editing are powerful tools for cancer immunotherapy. However, rapid degradation of nucleic acids by nucleases and their inadequate ability to internalize into the cells and crossing nuclear membrane are the main challenges [75]. Poly (b-amino esters), a cationic polymer that has been used extensively in gene delivery due to their high loading capacity and safety, was recently exploited to deliver the CRISPR-Cas9 genome editing system to knockout cyclin-dependent kinase 5 (*cdk5*) *in vivo* [76]. It was observed that deletion of *cdk5* downregulates PD-L1 and triggers strong T cell-mediated immune responses in TME. Consequently, tumor growth and metastasis are inhibited in 4T1 tumor-bearing mice [76].

Adenosine mediates immunosuppression within TME via binding its receptors on immune cells. Therefore adenosine and its receptors have been recognized as novel targets for cancer immunotherapy [77]. Masjedi et al. [78] demonstrated that targeting A2 adenosine receptor with siRNAs loaded into nanoparticles enhances the production of inflammatory cytokines and triggers activation of T cells in the 4T1 breast tumor-bearing mice. Increased production of adenosine within the TME was observed as a result of overexpression of CD39 and CD73. Therefore, targeting CD39 and CD73 could be another strategy for cancer treatment. Administration of CD73 siRNA-loaded chitosan-lactate nanoparticles with tumor lysate pulsed DCs vaccine showed a great antitumor efficacy in 4T1 breast cancer bearing mice [79].

### Targeting molecules involved in TME formation by nanoparticles

As discussed earlier, TME plays a critical role in cancer development and progression, and thus emerged as promising target for antitumor treatment. It has been proven that acidic TME can affect CTL activity. Lactate dehydrogenase A plays a major role in tumor acidity by converting pyruvate to lactic acid in tumor cells. It was reported that delivery of siRNAs targeting lactate dehydrogenase A by cationic lipid-assisted nanoparticles in 4T1 mammary tumor-bearing mice neutralizes tumor pH, elicits infiltration of CD8^+^ T and NK cells and impedes the growth of tumors [80]. Moreover, the concentration of copper within the TME is also an important factor in controlling tumor growth [81]. The amount of copper is highly elevated in breast carcinoma to assist tumor development and progression through angiogenesis. Inhibition of human copper trafficking remarkably lessens tumor cell proliferation. Zhou and colleagues [82] constructed a copper chelating coil-comb block copolymer loaded with resiquimod (a TLR7/8 agonist), and demonstrated that the developed complex hinders mobility, invasion and vascular tube formation of human umbilical vein endothelial cells via copper chelation and promotes maturation and activation of human plasmacytoid DCs, leading to a marked inhibition of both primary breast tumor and lung metastases [82].

Overall, current evidence supports that nanoparticle-mediated immunotherapy represents a viable and attractive strategy for the treatment of breast cancer.

## Nanoparticle-mediated combination therapy in breast cancer

A combination of cancer immunotherapy with other conventional therapeutic strategies such as chemo-, photothermal-, and photodynamic-therapy has demonstrated an outstanding efficacy in the treatment of various types of cancers, including breast cancer. Nanoparticles allow for a practical and safe combination of these treatments.

### Combination with chemotherapy

Lei and co-workers [83] reported that a combination therapy of avasimibe (a small molecule inhibitor of acyl-coenzyme A-cholesterol acytransferase-1 that can strengthen CD8^+^ T cell activity) and a nano-drug delivery system containing doxorubicin generates a better efficacy in tumor growth inhibition compared with monotherapy in 4T1 tumor‐bearing mice. Consistent with this finding, doxorubicin-polyglycerol-nanodiamond conjugate was found to be more effective in reversing immunosuppression of the TME to achieve better therapeutic outcomes as compared to free form of doxorubicin in the 4T1 mouse model [84]. Moreover, co-application of doxorubicin-loaded micelles with imiquimod-loaded micelles was observed to trigger strong CTL responses towards 4T1 orthotopic tumor in mice and significantly diminish tumor growth and metastasis [85]. Liu et al. [86] designed a nanomedicine consisting of curcumin (a natural antitumor compound found in the spice turmeric)-loaded polymeric nanoparticles and a nanovaccine containing CpG and antigenic peptides. After injection in 4T1 breast cancer model, this nanomedicine efficiently triggers immunogenic cell death (ICD) of cancer cells and activation of DCs. In addition, release of immunostimulatory agents from nanovaccine in tumor sites assists in the stimulation of DCs, causing a significant improvement in tumor-specific CD8^+^ T-cell response. This combination induces a strong tumor-specific CD8^+^ T-cell responses that significantly inhibit tumor growth [86]. Together, these studies suggest that combining immunotherapy with chemotherapy via nanomedicines offers a strategy for better treatment of breast cancer.

### Combination with photothermal therapy (PTT)

PTT and photodynamic therapy (next section) are relative safe; however, they cannot eradicate primary tumor and their therapeutic efficacy against metastasis and recurrence is also ineffective. To address this challenge, a layered double hydroxide nanoparticle carrying three therapeutic drugs (i.e., indocyanine green as a photothermal agent, doxorubicin, and CpG) was constructed [87]. This multifunctional nanoparticle was proven to be very effective in eradicating primary tumor and preventing tumor recurrence and metastasis in the 4T1 breast cancer mouse model [87]. Similar results were obtained on a nanoparticle that integrates IR 820 as a photothermal agent and glycol chitosan as an immunostimulatory agent [88]. Albumin is the most abundant protein in plasma and has been employed to design biomimetic nanoparticles. A mitochondria-targeted photochemo-therapeutic nanoparticle was recently constructed by incorporating doxorubicin and triphenylphosphonium-tailored IR780 derivative into bovine serum albumin [89]. Mitochondria lack DNA repair process and are considered as a favorable target for doxorubicin-mediated chemotherapy in multidrug resistance cells. It was shown that targeting mitochondria with this nanoparticle induces strong ICD and T cell immune responses, leading to significant inhibition of tumor growth, metastasis and recurrence in the 4T1 mouse tumor model [89].

Gold nanoparticles have the ability to initiate photothermal ablation after irradiation with a near-infrared laser. Single injection of gold nanoparticles loaded with an anti-PD-1 peptide and subsequent irradiation at the tumor site demonstrated an excellent antitumor effect against breast cancer [90]. Similarly, treatment with bovine serum albumin coupled with gold nanorods showed strong immune-stimulatory responses of DCs and tumor suppression [91]. Fe_3_O_4_ nanoparticles coupled with reduced-graphene oxide have also been revealed to serve as an excellent platform for photothermal-immunotherapy. After laser irradiation, these nanoparticles induce ICD and DC activation, and destroy the primary tumors in the 4T1 mouse model [92]. Additionally, Wang et al. [93] developed a modified copper sulfide nanoparticle that can induce photothermal effects. After in vivo delivery of this copper-based nanoparticle in combination with an anti-PD-L1, they observed a strong inhibition of the growth of both primary and distant tumors in the 4T1 tumor model due to an induction of systematic immune responses.

### Combination with photodynamic therapy (PDT)

PDT induces the production of reactive oxygen species (ROS), which can trigger ICD and DC activation. Liu and coworkers [94] formulated a serum albumin-coated nanoparticle for delivery of photosensitizer chlorin e6 and honey bee venom melittin peptide to 4T1 tumor-bearing mice. They found treatment with this nanoparticle promotes the production of ROS, leading to enhanced APC stimulation and tumor cell lysis [94]. Core–shell nanoparticles developed using polymer of Zn and pyrophosphate have the advantages of high-profile safety, prolonged blood circulation, and minimal uptake by the mononuclear phagocyte system due to the existence of endogenous Zn and pyrophosphate in blood plasma. Addition of pyrolipid as a nontoxic photosensitizer demonstrated its effectiveness in killing breast cancer cells after laser irradiation and sensitizing tumors to immune checkpoint inhibitors in the 4T1 murine model [95]. Moreover, Marrache et al. [96] utilized the mitochondria-targeted nanoparticles to deliver the zinc phthalocyanine photosensitizer to cancer cells and demonstrated a substantial efficacy in breast cancer treatment [97]. In a similar study, another photosensitizer named photochlor was loaded into a nanoparticle, which was revealed to trigger strong host antitumor immunity, thereby suppressing tumor growth and metastasis in the 4T1 murine breast cancer model [96]. It was also reported that addition of an antigen-capturing agent maleimid to the photothermal/phototherapeutic-based nanoparticles significantly enhances antigen presenting to DCs following ICD caused by the nanoplatform and impedes both primary and distant tumors in the 4T1 tumor model [98].

It is recognized that induction of cancer cell pyroptosis can remarkably boost immune system against various tumors. Recent studies demonstrated that pyroptosis-based chemo- or phototherapy also provides effective strategy for inhibition of both primary and distant tumor growth, as evidenced by that activation of caspase-3 and upregulation of gasdermin E (a pyroptosis-inducing molecule) via photo- or chemotherapeutic-based nanoparticles trigger pyroptosis, consequently eliciting strong immune responses and tumor inhibition in breast tumor-bearing mice [99, 100].

Altogether, these combination therapies have shown satisfactory outcomes in breast cancer treatment. Merging these therapies through nanoparticles offers the possibility of killing both primary and distal tumors most reliably.

### Simultaneous delivery of multiple antitumor agents via a nanoparticle

Development of multifunctional nanoparticles that are able to carry different therapeutic agents is of interest. In line with this, a dual pH-responsive multifunctional nanoparticle was developed by attachment of poly (L-histidine)-resiquimod complex with hyaluronic acid-doxorubicin conjugate to deliver resiquimod and doxorubicin simultaneously. Ionization of poly (L-histidine) at TME (~ pH 6.5) leads to the release of resiquimod, followed by activation of APCs, while disruption of hydrazone bond in hyaluronic acid-doxorubicin at endo/lysosomes (~ pH 5.5) results in the release of doxorubicin in cancer cells, thereby causing a cooperative antitumor effect in 4T1 tumor-bearing mice [101]. Polypyrrole is an organic nanostructure, widely applied as a photothermal agent. Camptothecin is a traditional Chinese medicine with cytotoxic activities against cancer cells. Encapsulation of both therapeutic agents in hyalurunic acid generates a robust tumor-targeted therapeutic platform, leading to effective ICD in breast cancer cells [102]. Furthermore, it was shown when combined with PD-L1 inhibitors, this fabricated complex provokes strong antitumor immune responses, resulting in eradication of primary breast cancer and suppression of tumor metastases and recurrences in 4T1 tumor-bearing mice [102].

To improve immunotherapy, Feng et al. [103] formulated a binary cooperative prodrug nanoparticle loaded with chemotherapeutic oxaliplatin and NLG919 (a regulator of indoleamine 2,3-dioxygenase 1, which plays a key role in immunosuppression) and described a synergistic antitumor effect in comparison with free oxaliplain and/or free NLG919 in mouse models of both breast and colorectal cancer. Poly vinyl alcohol is an FDA-approved chemical that is extensively exploited in nanotechnology. Its modification with vinyl ether acrylate groups exhibits great biocompatibility, safety and acidic degradation property. Qiao and coworker [104] fabricated a poly vinyl alcohol-based nanogel loaded with both docetaxel and an indoleamine 2,3-dioxygenase 1 inhibitor. Compared with free drug, application of this nanogel was shown to induce higher antitumor immunity and stronger tumor inhibition effect in 4T1 mouse breast cancer model.

Cancer stem cells play a key role in cancer recurrence and resistance to chemo-immunotherapy. To overcome this drawback, Lang et al. [105] developed a micelle-liposome double-layer particle to encapsulate anti-cancer stem cell agent thioridazine, together with paclitaxel and a PD-1/PD-L1 inhibitor and demonstrated a significant inhibition of tumor growth in mice bearing metastatic MCF‐7 tumors. Finally, Arg-Gly-Asp peptide-modified lipophilic prodrugs were recently employed for targeted delivery of paclitaxel and imiquimod to breast cancer cells. It was shown that infiltration and activation of CTLs could directly destroy cancer cells and prevent tumor metastasis and recurrence in 4T1 tumor model [106].

## Other novel types of nanoparticles

Besides diverse types of nanocomplexes as discussed above, virus-like nanoparticles, referring to as noninfectious protein shells, or capsids of the virus without virus genome, are broadly exploited for effectual cancer immunotherapy as they can generate strong immune responses. Virus-like nanoparticles can be used either alone or with diverse immunostimulatory molecules. It was shown that inhalation of self-assembling virus-like nanoparticles from the cowpea mosaic virus elicits significant systematic immune responses and tumor inhibition in different tumor models, including 4T1 breast cancer model [107]. Viral capsid VP2 protein of the human parvovirus B19 has also been developed as virus-like particles to deliver neoepitopes to increase cellular immunity [108, 109]. Chimeric VP2 was generated to display epitopes from Tmtc2, Gprc5 Oars and survivin [108] or insulin-like growth factor-1 receptor [109]. Following assembly, administration of these virus-like particles was found to significantly suppress tumor growth and lung metastasis, as compared to treatment with epitopes or native VP2 alone, in the 4T1 breast cancer model [108, 109].

In addition to virus-like nanoparticles, phage nanoparticles have been exploited to improve antitumor immunogenicity in breast cancer. For instance, peptides derived from HER2 proteins were fused with the lambda phage (λF7) coat protein gpD to generate vaccines, which induce powerful antitumor immunity against HER^+^ TUBO breast cancer in mice [110, 111].

**Fig. 3:**
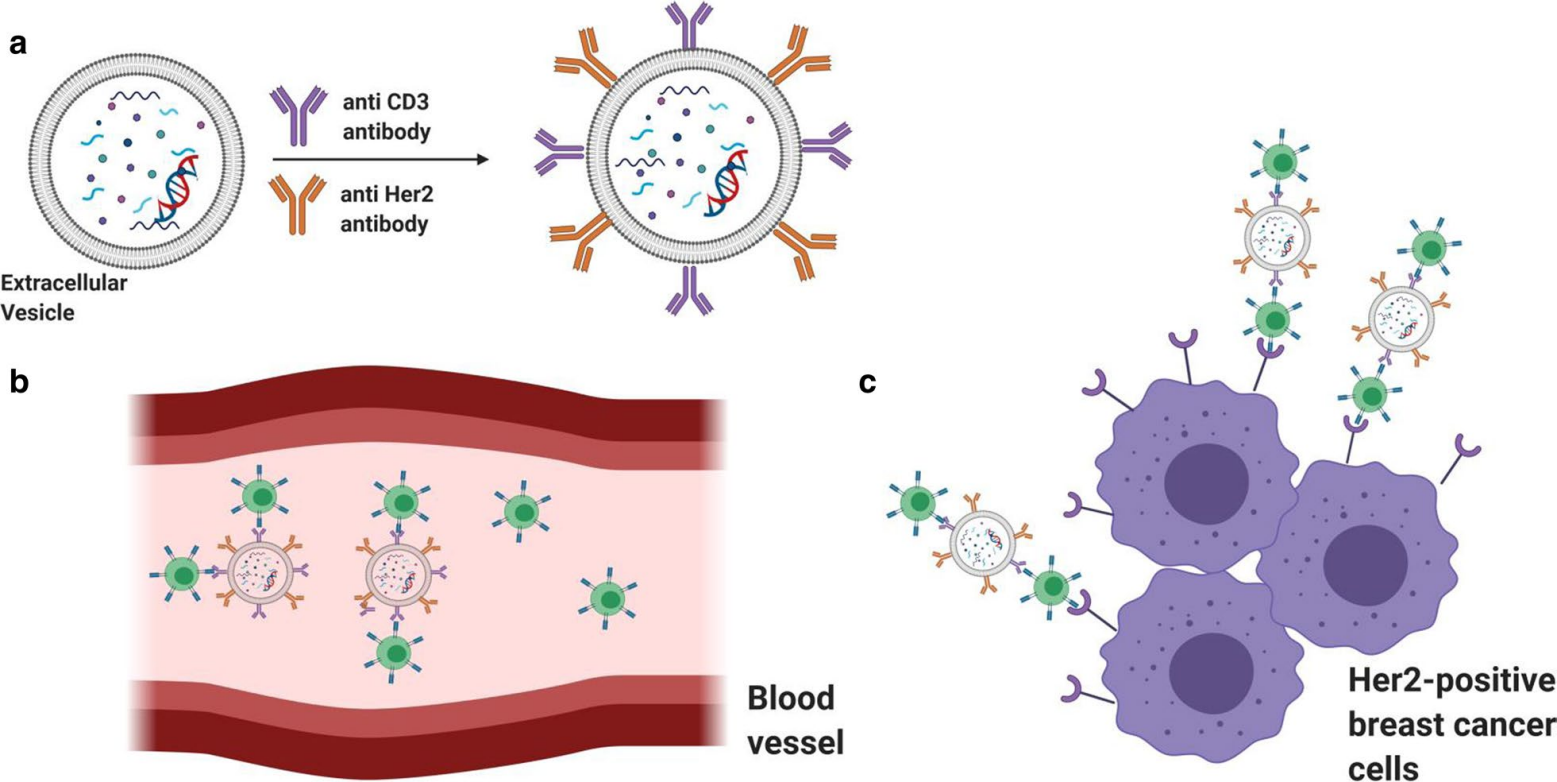
Using extracellular vesicles for efficient breast cancer immunotherapy. **a** Adding anti CD3 and anti Her2 antibodies on the surface of extracellular vesicles. **b** Binding of engineered extracellular vesicles to the CD3-positive T cells in blood circulation. **c** Recruitment of T cells to tumor microenvironment, consisting of the Her2-positive tumor cells through interaction of T cells-extracellular vesicles complex with Her2-positive cells

Extracellular vehicles (EVs) were also widely used for cancer treatment. EVs are generally safe with minimal toxicity and off-target impacts after in vivo administration. It was shown that EVs isolated from NK cells are able to destroy cancer cells in vitro and in vivo through multiple injection methods [112]. Further study by the same group revealed that EVs derived from NK cells pre-exposed to IL-15 have stronger antitumor potency compared with EVs isolated from NK cells with no pretreatment [113]. NK cell membrane was also used for formation of nanoparticles and showed good immune activity in the TME [114]. In addition, genetically engineering EVs is a possible way to add molecules to them either for therapeutic or targeting purposes. For example, EVs were genetically engineered to express anti-human CD3 and anti-human HER2 antibodies. This modification allows EVs to specifically target CD3 and HER2 receptors on T cells and HER-positive breast cancer cells, respectively, leading to the recruitment of myriad CTL to TME and a significant tumor growth inhibition (Fig. 3) [115].

## Conclusion

Due to high heterogeneity, treatment of breast cancer remains challenging, especially for patients suffering from TNBC. Immunotherapy has emerged as a promising treatment approach compared to other conventional modalities and countless efforts have been made to develop powerful immunostimulatory agents to boost immune responses. Using such therapeutic agents without modifications has encountered limitations such as rapid clearance and off-target delivery. Nanoparticle-based immunotherapy holds great potential to overcome these limitations. Up to now, a growing body of evidence has proven excellent effectiveness and safety with nanoparticle‐mediated cancer immunotherapy in comparison with current immunotherapy strategies. Most excitingly, in addition to the pre-clinical studies described in this review, several clinical trials are undergoing to investigate the therapeutic efficacy of nanomedicine-mediated immunotherapy in advanced breast cancer. A phase I clinical trial has been initiated to evaluate the combination therapy of Atezolizumab (an anti-PD-L1 monoclonal antibody) and nanoparticle albumin-bound paclitaxel in patients with locally advanced or metastatic TNBC with PD-L1 positive (NCT04249167). Similar combination therapy has also been carried out in a phase II trial in patients with TNBC before surgery (NCT02530489). Another phase I/Ib study is undergoing to assess a combined therapy of Etrumadenant, an A2a and A2b adenosine receptor antagonist, with either pegylated liposomal doxorubicin or albumin-bound-paclitaxel in patients with locally advanced or metastatic TNBC (NCT03719326). All in all, nanoparticle‐based breast cancer immunotherapy is expected to find its way to clinical application as a promising therapeutic alternative to conventional therapies in the near future.

## Data Availability

Not applicable.
